# Detection of Double Compression in HEVC Videos Containing B-Frames

**DOI:** 10.3390/jimaging11070211

**Published:** 2025-06-27

**Authors:** Yoshihisa Furushita, Daniele Baracchi, Marco Fontani, Dasara Shullani, Alessandro Piva

**Affiliations:** 1 Department of Information Engineering, University of Florence, 50139 Firenze, Italy; daniele.baracchi@unifi.it (D.B.); dasara.shullani@unifi.it (D.S.); alessandro.piva@unifi.it (A.P.); 2 Amped Software, 34149 Trieste, Italy; marco.fontani@ampedsoftware.com

**Keywords:** video forgery, double compression, H.265/HEVC, B-frames

## Abstract

This study proposes a method to detect double compression in H.265/HEVC videos containing B-frames, a scenario underexplored in previous research. The method extracts frame-level encoding features—including frame type, coding unit (CU) size, quantization parameter (QP), and prediction modes—and represents each video as a 28-dimensional feature vector. A bidirectional Long Short-Term Memory (Bi-LSTM) classifier is then trained to model temporal inconsistencies introduced during recompression. To evaluate the method, we created a dataset of 129 HEVC-encoded YUV videos derived from 43 original sequences, covering various bitrate combinations and GOP structures. The proposed method achieved a detection accuracy of 80.06%, outperforming two existing baselines. These results demonstrate the practical applicability of the proposed approach in realistic double compression scenarios.

## 1. Introduction

As video viewing on smartphones and tablets increases and 4K and 8K TVs become more widespread, the demand for higher-definition and clearer video has grown. As a result, there is a need for new encoding technologies that can efficiently transmit large video data over limited network bandwidth while maintaining image quality. The next-generation video coding standard, H.265/HEVC (HEVC) [1,2], was developed to address this challenge. HEVC employs compression techniques similar to its predecessor, H.264/AVC (AVC) [3]. However, it introduces improvements such as increasing coding block size, adding more intra-prediction directions, efficiently encoding motion vectors, and enhancing inter-prediction efficiency. These improvements allow HEVC to achieve approximately twice the compression rate compared to AVC.

Moreover, the rise of video editing on mobile devices and the availability of affordable and user-friendly tools have made it easy for anyone to edit original videos, complicating verifying their authenticity and reliability. As a result, the importance of video tampering detection technologies has increased. Verifying the integrity of videos is especially crucial when they are submitted as legal evidence.

Video tampering often involves a process known as double compression, where a video is decoded, edited, and then recompressed. Since an original video should only be encoded once when recorded, detecting double compression is a key indicator that the video is not a camera-original file, calling for further investigation to understand whether evidence was handled the wrong way, leading to unnecessary re-compression and data loss, or if some possibly malicious tampering was carried out. In multimedia forensics, various methods have been developed to detect double compression.

Wang et al. demonstrated that when a video is recompressed after frame deletion or insertion, periodic prediction errors occur because the video is encoded with a different Group of Pictures (GOP) structure than the original [4]. Based on this, Stamm et al. extended the research and proposed a method to detect frame deletion or insertion automatically [5]. When the GOP structure changes between the first and second encoding processes, frames originally encoded as I-frames may be re-encoded as P- or B-frames, resulting in significant differences from the original P- or B-frames. Refs. [6,7] utilized the re-encoded frames, and they referred to them as “relocated I-frames (RI-frames)”. Vázquez-Padín et al. analyzed anomalies in macroblock types within RI-frames in double-compressed videos and named this characteristic “Variation of Prediction Footprints (VPF)” [8], and they later extended their research to consider B-frames [9]. However, these studies focused on videos encoded with AVC or MPEG and may not apply to videos encoded with HEVC.

Research on detecting double compression in HEVC videos can be classified into two categories: “aligned GOP,” where the GOP structure used in the first and second encoding processes is the same, and “non-aligned GOP,” where the GOP structure differs between the two encodings. In the case of non-aligned GOP, it is necessary to consider RI-frames. While these classifications have been widely explored, existing methods primarily focus on I-frames and P-frames, overlooking the distinct behavior of B-frames in double-compressed HEVC videos. B-frames, which utilize both forward and backward prediction, present unique compression artifacts that are not captured by previous approaches.

Researchers have proposed various methods for the case of aligned GOP to identify double-compressed videos. For instance, SVM-based methods have been developed that use the co-occurrence matrix of DCT coefficients or the distribution of Transform Unit (TU) size and DCT coefficients, which vary with the quantization parameter (QP), to detect double compression [10,11,12]. Other approaches include using compression idempotency to identify transcoded HEVC videos [13] and detecting double-compressed videos that have been re-compressed with the same QP [14,15]. Additionally, histogram-based methods have been used to detect double compression by analyzing changes in the sizes of coding units, such as Prediction Unit (PU) and Transform Unit (TU) [16,17]. Some studies have observed that changes in encoding elements within I-frames are most pronounced between single- and double-compressed videos and tend to converge when additional compression is applied [18]. Other researchers have extracted features from intra-prediction in I-frames and inter-prediction in P-frames and B-frames to detect fake high-definition video [19].

In research on non-aligned GOP, several studies have utilized spatial inconsistencies observed in RI-frames and temporal inconsistencies with adjacent frames as features for detecting double compression [20,21,22,23]. Additionally, other methods have been developed to analyze the quality degradation process of double compression in non-aligned GOP structures and the decision mode of in-loop filtering [24]. In real-world scenarios, the GOP size set by the device that recorded the original video and the editing tools often differs. Therefore, the re-encoded video is expected to use a different GOP structure.

In [19], HEVC videos containing B-frames were re-encoded to detect fake high definition for HEVC videos. This study used an aligned GOP structure, making the second compression weaker than the first. In contrast, our research presents the first dedicated attempt to detect double compression in HEVC videos that contain B-frames, addressing a realistic and technically complex scenario that has not been explicitly tackled in previous work. By analyzing encoding behaviors unique to B-frames, our method offers new insights beyond the conventional focus on I/P-frame structures.

This paper is structured as follows. Section 2 overviews basic video encoding and the H.265/HEVC technology. Section 3 outlines the basic concept of relocated I-frames (RI-frames). Section 4 describes our proposed method, and Section 5 presents the experiments and results.

## 2. Preliminaries

This section overviews video encoding fundamentals, focusing on the H.265/HEVC standard [1,2]. Video encoding is a process that compresses raw video data to make it more efficient for storage and transmission while preserving as much quality as possible. H.265/HEVC, introduced as a successor to H.264/AVC, significantly improves compression efficiency by utilizing advanced techniques such as flexible block unit structures and enhanced prediction technology.

### 2.1. Basic Video Encoding

Video data consists of multiple frames categorized into three types: I-pictures, P-pictures, and B-pictures. An I-picture is typically the first frame in a GOP and is compressed independently without relying on other frames. Due to this independence, it generally has the largest data size and is a crucial reference point for subsequent frames. A P-picture typically references a preceding I-picture or another P-picture, compressing only the differences, which reduces its data size. A B-picture can reference both preceding and succeeding frames, making it more efficient and smaller in data size than a P-picture.

Frames are grouped into GOP structures, which contain a sequence of I-pictures, P-pictures, and B-pictures arranged in encoding order from one I-picture to the next. There are two types of GOPs: closed GOP and open GOP. In a closed GOP, all P-frames and B-frames within the GOP are decoded without referencing frames outside that GOP. In contrast, an open GOP allows frames within the GOP to reference frames from other GOPs for decoding. This study focuses solely on closed GOP, where P-frames and B-frames follow an I-frame within the same GOP. The current method is designed to specifically handle closed GOP structures due to the predictable reference patterns, simplifying feature extraction and analysis. Open GOPs introduce cross-GOP dependencies that require additional mechanisms for accurate analysis. Additionally, a sub-GOP refers to the sequence of consecutive B-frames between two reference frames within a GOP.

### 2.2. Basic Technology of H.265/HEVC

HEVC divides frames into blocks using four processing units for efficient encoding: Coding Tree Unit (CTU), Coding Unit (CU), Prediction Unit (PU), and Transform Unit (TU). CTU is the basic unit for partitioning an image, and each CTU consists of Coding Tree Blocks (CTBs) for luminance and chrominance components. It is further divided into variable-sized CUs based on recursive quad-tree partitioning. Each CU consists of Coding Blocks (CBs) for luminance and chrominance components and is further divided into variable-sized PUs and TUs responsible for prediction and transformation processing, respectively. The introduction of CTU, CU, PU, and TU allows the encoder to adjust processing according to the characteristics of the image and minimize prediction parameters, thereby reducing encoding costs. For example, in image regions with complex changes, many small CUs are allocated with more prediction parameters, such as motion vectors, to improve prediction performance. Conversely, large CUs are used for encoding regions with few changes, minimizing the number of prediction parameters.

In HEVC, intra-prediction is performed on luminance and chrominance signals to reduce redundancy and increase compression efficiency by taking advantage of the high correlation between adjacent pixels in an image. The Prediction mode selection is performed at the PU level, while quantization and de-quantization are performed at the TU level. Intra-prediction for the luminance component uses two standard prediction operators (DC and Planar) and 33 directional operators. Directional operators (2–34) predict a target pixel by referring to an encoded pixel at specified angles. Planar prediction (0) uses interpolated values from four adjacent pixels, while DC prediction (1) uses the average value of surrounding pixels. Intra-prediction assigns a mode to each PU, prioritizing the horizontal and vertical patterns commonly found in natural images. Prediction modes closer to these directions have smaller angular displacements than others.

Intra-prediction for chrominance components employs Planar (0), DC (1), horizontal (10), vertical (26), and Intra-derived modes (36). The Planar, DC, horizontal, and vertical prediction modes are explicitly signaled, but if they match the luminance intra-prediction mode, the angular prediction mode (34) is applied instead. In the Intra-derived mode (36), chrominance intra-prediction uses the corresponding luminance intra-prediction mode to reduce signal overhead for encoding.

In HEVC, as in H.264/AVC, motion compensation prediction is performed block-by-block. From reference frames stored in the frame memory, multiple motion vectors are searched from spatially and temporally adjacent PUs of the target PU for which the prediction vector will be determined. The motion vector with the smallest prediction error is selected from these options. While the expansion and diversification of block sizes have improved prediction efficiency, they have also introduced the drawback of over-segmenting the image compared to conventional methods, resulting in redundant signaling and inefficient boundaries. For example, adjacent blocks often share similar movements in regions with fast-moving objects or areas with clear motion vectors. However, in HEVC, child blocks belonging to different parent blocks cannot share motion information. To address this issue, HEVC employs the merge mode. This technique reduces encoding costs by reusing the motion information (motion vectors, reference picture indices, and prediction directions) of multiple adjacent encoded PUs and transmitting only the index of the PU. Additionally, skip mode is used for smooth image regions where prediction residuals can be ignored.

## 3. Basic Concept of RI-Frames

**Figure 1:**
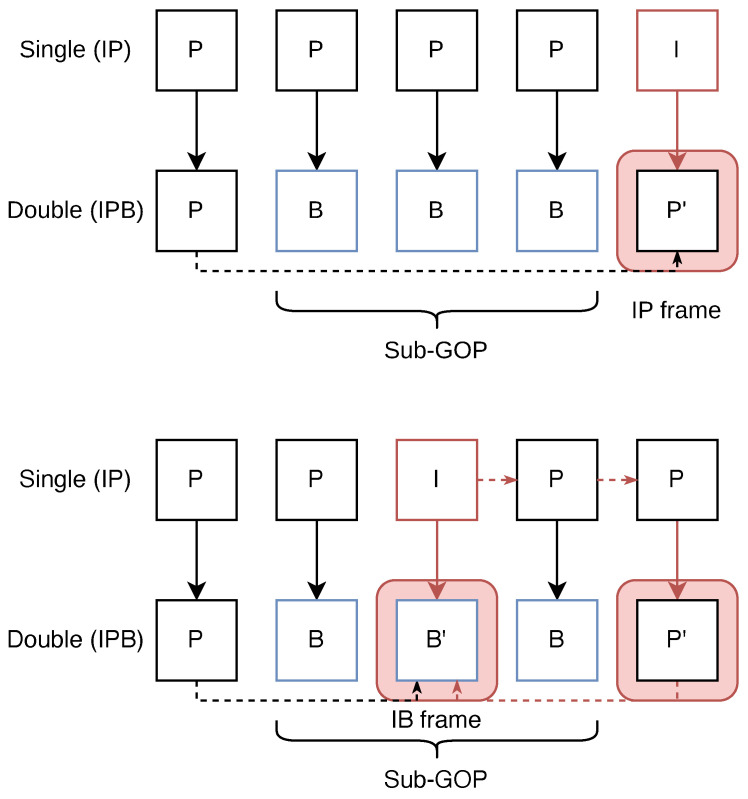
Basic concept of the RI-frames (top: IP, bottom: IB).

This study assumes that the first encoding process uses only I-frames and P-frames, while the second encoding process includes I-frames, P-frames, and B-frames. This assumption is reasonable, considering that the first encoding typically occurs during video acquisition, where B-frames are rarely used due to the extra computational burden they impose on live encoding systems. In the second encoding, the original I-frame may be re-encoded as a P-frame or B-frame. Figure 1 illustrates this process, showing how IP and IB frames are generated. In the figure, $P^{\prime }$ and $B^{\prime }$ represent frames influenced by the original I-frame from the first encoding.

IP and IB frames retain many features from the original I-frame during the second encoding. As detailed in [9], the quantization noise introduced during the first compression expands the scope of intra-prediction, and the selection of reference frames during re-encoding reduces inter-prediction accuracy. As a result, the proportion of intra-prediction increases, while inter-prediction decreases in IP and IB frames. In the case of IB frames, consecutive B-frames within a sub-GOP refer to the preceding P-frame and the subsequent $P^{\prime }$ frame for bidirectional prediction. However, in the first encoding, the P-frames following the I-frame were predicted by referencing the I-frame, and the elements of the I-frame propagated to the subsequent P-frames. The $P^{\prime }$ frame, immediately following the sub-GOP, is a re-encoded version of the P-frame containing elements of the I-frame, and IB frames tend to reference the motion information from this $P^{\prime }$ frame.

## 4. Proposed Method

**Figure 2:**
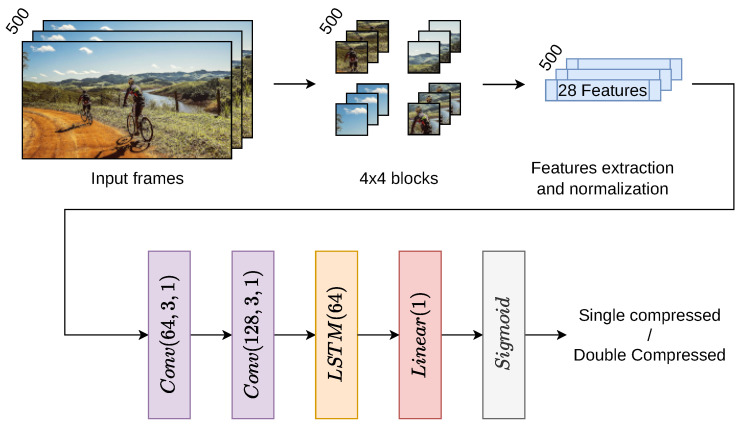
Flowchart of the proposed method.

This study proposes a method to detect double compression in HEVC videos by analyzing frame-level encoding information and using a bidirectional Long Short-Term Memory (Bi-LSTM) classifier. Our approach is to divide each frame into blocks, extract encoding information, generate feature vectors, and feed them into the LSTM model. Figure 2 visually represents the method’s workflow, from input processing to classification.

We performed feature extraction for each frame to identify abnormal behaviors in IP and IB frames in single- and double-compressed videos, as described in Section 3. The feature vector used in this analysis consists of elements shown in Table 1, represented as a 28-dimensional vector per frame, resulting in a final feature vector of 28×N dimensions, with *N* being the number of frames analyzed in the video.

To justify the choice of the 28-dimensional feature vector, we conducted an ablation study during the experimental phase. Starting from an 80-dimensional feature set composed of all available features, we systematically reduced the dimensionality by removing specific feature groups such as quantization parameters or certain intra-prediction directions. Each configuration was evaluated using 10-fold cross-validation, and the 28-dimensional feature vector—consisting of frame type, CU type, CU size, and selected luminance and chrominance prediction directions—consistently yielded the highest classification accuracy with the lowest standard deviation.

In all configurations, Frame Type and CU Type were retained, as they provide fundamental structural and motion-related information. The remaining features, such as QP, CU Size, Luminance, and Chrominance prediction directions, were selectively included or excluded to assess their contribution, as shown in Table 2.

The 28-dimensional feature vector, which excludes QP and includes CU Size along with selected prediction directions, consistently achieved the highest classification accuracy (80.06%) and the lowest standard deviation (1.16%), as also reported in Table 2. This result demonstrates an effective trade-off between information richness and feature redundancy.

First, we examine the frame type of each frame, followed by analyzing CU size variations. For P-frames, CU types are classified as intra-coded (PI), skipped (PS), merged (PM), inter-coded with zero motion vectors (PVZ), and inter-coded with non-zero motion vectors (PVNZ). For IB frames, CUs are classified as intra-coded (BI), skipped (BS), and merged (BM), with inter-coded CUs further categorized as past (BP), bidirectional (BB), or future (BF). Subsequently, we extract luminance and chrominance prediction directions for intra-prediction.

In the following sections, we present a detailed statistical analysis of encoding characteristics, including IP and IB frame behaviors in both single- and double-compressed HEVC videos. Additionally, we provide an in-depth description of the LSTM model used for classification.

### 4.1. Analysis of Extracted Encoding Elements

Using HM 16.25 [25], each frame was divided into 4 × 4 blocks, and encoding information such as CU size, CU type, and luminance and chrominance intra-prediction directions was extracted from the decoder output. The features listed in Table 1 are not computed via analytical formulas but are directly obtained from the decoder trace. Each element was then encoded as follows:

- Frame type was identified from the frame header and manually one-hot encoded in our study as I = (1, 0, 0), P = (0, 1, 0), and B = (0, 0, 1).
- CU size was parsed from the CU partitioning information in the decoder log and assigned an index according to block sizes: 64 × 64, 32 × 32, 16 × 16, and 8 × 8.
- CU type (Intra, Skip, Merge, Inter) was determined based on the prediction mode for each CU and categorized with corresponding index values.
- Intra-prediction directions for both luminance and chrominance were extracted and encoded using predefined index values based on the direction mode.

**Figure 3:**
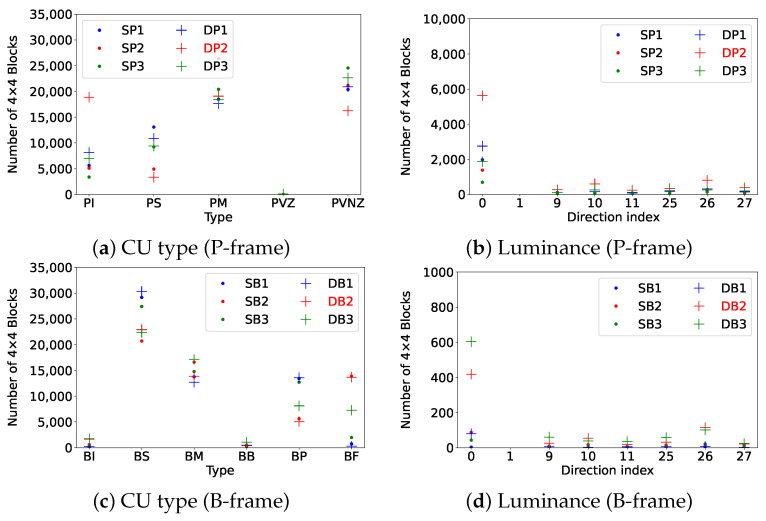
Encoding element comparison between single- and double-compressed videos. IP (DP2) or IB (DB2) frames are depicted in red. The values represent averages across 387 single-compressed and 1161 double-compressed videos.

Figure 3 compares the distribution of CU types and luminance intra-prediction directions in three consecutive P-frames and B-frames within a sub-GOP in single- and double-compressed videos.

In this analysis, we constructed a dataset comprising 129 videos in total. Specifically, the dataset includes 43 original YUV videos, 43 videos processed with BLUR, and 43 videos processed with CLAHE [26]. The single-compressed videos were encoded using a GOP size of 25, a sub-GOP size of 3, and bitrates of 1000, 3000, and 5000 kbps. For the double-compressed videos, the initial encoding used only I- and P-frames, with a GOP size of 12 and the same three bitrates. The subsequent compression employed the same parameters (GOP size, sub-GOP size, and bitrates) as those used for the single-compressed videos. This setup resulted in a total of 387 (3 × 129) single-compressed data points and 1161 (3 × 3 × 129) double-compressed data points for analysis.

In Figure 3, the top-left graph presents the distribution of CU types in P-frames, where an increase in the proportion of intra-coded CUs (PI) can be observed in double-compressed videos compared to single-compressed videos, while the proportions of skip-coded CUs (PS) and intra-coded CUs with non-zero motion vectors (PVNZ) decrease. This rise in PI results in more pixel values being used for luminance prediction.

In this analysis, SP1, SP2, and SP3 represent the first, second, and third P-frames, respectively, within the sub-GOP, where SP2 corresponds to the IP frame. Similarly, SB1, SB2, and SB3 represent the first, second, and third B-frames, where SB2 corresponds to the IB frame. Furthermore, SP and SB denote single-compressed P-frames and B-frames, respectively, while DP and DB denote double-compressed P-frames and B-frames. The frame numbering (e.g., DP1, DP2, DP3 for double-compressed P-frames and DB1, DB2, DB3 for double-compressed B-frames) is consistent with their single-compressed counterparts (SP and SB), reflecting the same positional structure within the sub-GOP. In order to simplify the explanation, only sub-GOPs where the IP or IB frame is located at position 2 were considered; therefore, both IP and IB frames are always positioned at the center of their respective sequences in this analysis.

The bottom-left graph displays the distribution of CU types in B-frames. In double-compressed videos, the proportion of intra-coded CUs (BI) rises to alter the initial IB frame, while skip-coded CUs (BS) and inter-coded CUs with past motion vectors (BP) decrease, and inter-coded CUs with future motion vectors (BF) increase in the third frame. This behavior is in line with what was reported by Vazquez et al. [9], where prediction errors introduced during the first encoding are compensated by an increased proportion of intra-coded components during re-encoding.

Additionally, the P-frame following the sub-GOP exhibits characteristics similar to the IB frame, resulting in increased inter-prediction dependency, which leads to a decrease in BP and an increase in BF. Furthermore, increased intra-prediction leads to higher luminance and chrominance values.

### 4.2. Model Architecture

The feature vectors are fed into a Bi-LSTM classifier, effectively capturing forward and backward dependencies in sequential data. This architecture allows the LSTM to detect both spatial anomalies within and temporal anomalies between frames. Traditional neural networks, such as fully connected or convolutional networks, lack mechanisms to retain temporal information, making them less suited for sequential data modeling. In contrast, the Bi-LSTM is well-suited for identifying compression-induced changes between frames, offering high robustness in detecting double compression (see Table 3 for the detailed layer configuration). To ensure consistent scaling, Min-Max normalization was applied, adjusting each feature to a range of 0 to 1. Model performance was evaluated using cross-validation on the training set, and the model with the highest cross-validation accuracy was selected to evaluate the test data.

To implement our double compression detection, we designed a Bi-LSTM classifier that combines CNN and LSTM layers (the complete configuration is summarized in Table 3). First, the model utilizes two one-dimensional convolutional layers, where the input is processed to extract local features. The first convolutional layer has 28 input channels and 64 output channels, with a kernel size of 3 and a stride of 1, while the second layer has 64 input channels and 128 output channels, also with a kernel size of 3 and a stride of 1. Both convolutional layers are followed by a ReLU activation function, adding non-linearity and enhancing the model’s capacity to capture complex feature patterns.

The extracted feature maps are then passed through a Bi-LSTM composed of two layers designed to capture temporal dependencies in the encoding information. The LSTM has an input size of 128 and a hidden layer size of 64, allowing it to analyze sequential frame data from both forward and backward directions effectively.

Finally, the output from the LSTM is processed through a linear layer that maps the 64-dimensional hidden state to a single output. A sigmoid activation function follows, restricting the output to the range [0, 1], which enables the model to perform binary classification with probabilistic output, identifying whether the input is double-compressed.

The initial hyperparameter settings—such as the number of convolutional filters, kernel size, and LSTM hidden size—were informed by He et al. [23], who proposed a hybrid architecture combining attention-based ResNet modules and LSTM layers for double compression detection in HEVC videos. While our architecture differs in structure and input representation, their use of LSTM to capture temporal dynamics in the compression domain guided the configuration of our recurrent layers. We then empirically fine-tuned these parameters based on validation performance. The final model contains approximately 228,865 trainable parameters, which is reasonably lightweight and appropriate given the dimensionality of the input features and the available training data.

## 5. Experimental Results

This section describes the experimental validation of the proposed model. We detail the dataset creation process and compare the accuracy of our double compression detector with the state-of-the-art methods. Bitrate and GOP size are crucial factors in determining video quality, so we constructed the dataset using various combinations of these parameters. Additionally, we analyzed how these two encoding parameters affect model performance and examined the impact of reducing the number of frames in the test data. For evaluation, single-compressed videos are labeled as negative, and double-compressed videos are labeled as positive, ensuring no overlap in video content between the training and test datasets.

### 5.1. Dataset

We used 43 YUV videos (720p: ducks_take_off, FourPeople, in_to_tree, Johnny, KristenAndSara, mobcal, old_town_cross, park_joy, parkrun, shields, sintel_trailer, stockholm, vidyo1, vidyo3, vidyo4. 1080p: aspen, controlled_burn, crowd_run, dinner, factory, life, red_kayak, rush_field_cuts, rush_hour, snow_mnt, speed_bag, sunflower, touchdown_pass, tractor, west_wind_easy. 4K: Netflix_Aerial, Netflix_BarScene, Netflix_DinnerScene, Netflix_Dancers, Netflix_DrivingPOV, Netflix_FoodMarket, Netflix_PierSeaside, Netflix_RitualDance, Netflix_RollerCoaster, Netflix_SquareAndTimelapse, Netflix_ToddlerFountain, Netflix_TunnelFlag, Netflix_WindAndNature.) (720p, 1080p, 4K) [27]. Each video retained up to the first 500 frames, and the classifier was designed with a base resolution of 720p. However, because the classifier processes a 28-dimensional feature vector per frame, it is resolution-independent and can handle videos of any size. The scale of the feature vector remains consistent across resolutions due to normalization, enabling the processing of videos in various sizes without resizing or padding.

For data augmentation, we used FFmpeg (https://www.ffmpeg.org/, accessed on 19 June 2025) to extract frames from the YUV videos and applied CLAHE [26] and Blur processing using the albumentations library (https://pypi.org/project/albumentations/, accessed on 19 June 2025). We set the probability parameter *p* = 1.0, ensuring that blur is applied to every frame. The blur was implemented as a uniform box blur with a randomly selected square kernel size between 3 and 7 pixels, with larger kernels producing stronger blur effects. Each frame was processed sequentially with these augmentation methods and then recombined into YUV format using FFmpeg. This process resulted in three subsets of YUV videos for each original video content: the original (O), CLAHE-processed (C), and blur-processed (B) videos. These three subsets are derived from a single YUV video and are, therefore, grouped as a single dataset. This approach prevents content duplication by treating all subsets from the same source video as one dataset unit.

Table 4 summarizes the encoding parameters used for single and double compression. Single-compressed videos were encoded using x265 with bitrates B2 (1000, 3000, 5000 kbps), GOP sizes G2 (9, 25, 70), and sub-GOP sizes SG (3, 5, 7). For double compression, videos were first encoded with only I- and P-frames at bitrates B1 (1000, 3000, 5000 kbps) and GOP sizes G1 (12, 30), then re-encoded with the same parameters (B2, G2, SG) as in single compression. HM 16.25 was used to extract the encoding information. For encoding with x265, closed GOP (–no-open-gop encoding flag) was used to ensure that each GOP referenced only its own frames, creating a uniform prediction structure. Additionally, B-frames were set to reference only I- or P-frames, disabling references to other B-frames (–no-b-pyramid encoding flag), simplifying prediction. Each dataset (O, C, B) contains 81 single-compressed videos (3 × 3 × 3 × 3) and 486 double-compressed videos (3 × 2 × 3 × 3 × 3). To ensure a fair evaluation, 81 double-compressed videos were randomly selected.

For all performance evaluations, 40 video datasets were randomly selected from the original 43 datasets. We applied 10-fold cross-validation, using 32 sets for training, 4 for validation, and 4 for testing. All experiments were performed on a machine equipped with an NVIDIA RTX 2080 Ti GPU, 32 GB of RAM, and an AMD Ryzen 9 5900X CPU (Advanced Micro Devices, Santa Clara, CA, USA).

### 5.2. Performance Evaluation on Double Compression Detection

The proposed model, implemented in PyTorch (version 2.3.1+cu118), was optimized using the Adam algorithm with momentum parameters $\beta_{1}=0.9$ and $\beta_{2}=0.999$. A mini-batch size of 64 and an initial learning rate of 0.005 (decayed by γ=0.5 every 6 epochs) were employed, up to a maximum of 50 epochs. Two Bi-LSTM layers enhanced feature extraction, and early stopping was applied if the validation loss did not improve for 10 consecutive iterations.

In the evaluation, we compared our method against two well-known approaches to the double compression detection problem and reported the accuracy performances in Table 5. The first one [9] was originally developed for H.264/AVC videos, where it utilizes macroblock (MB) types as input and analyzes MB behavior based on multiple rules. Since HEVC uses coding units (CUs) instead, we modified the input to utilize CU data extracted from each frame.

The second one, proposed in [23], was designed for HEVC videos, but it does not consider B-frames and focuses only on variations in CU size and type over three consecutive P-frames. However, since our dataset includes both P- and B-frames, we adapted their method to operate using only P-frames, allowing for a fair comparison under our experimental setup.

Table 5 shows the average performance of ten evaluations, each based on 10-fold cross-validation with a different random seed. For evaluation, we considered the true positive rate (TPR), true negative rate (TNR), and balanced accuracy (ACC). TPR, indicating the proportion of actual positive samples correctly predicted, was calculated as $\mathrm{TPR}=\frac{\mathrm{TP}}{\mathrm{TP}+\mathrm{FN}}$, while TNR, representing the proportion of actual negative samples correctly predicted, was calculated as $\mathrm{TNR}=\frac{\mathrm{TN}}{\mathrm{TN}+\mathrm{FP}}$. Balanced accuracy was then computed as $\mathrm{ACC}=\frac{\mathrm{TPR}+\mathrm{TNR}}{2}$. Standard deviation (SD) was computed across the ten balanced accuracy values, reflecting the stability of each method under different data splits.

At the considered working point, the proposed method outperforms He et al. [23] in terms of True Negative Rate (TNR) and Vazquez et al. [9] in terms of True Positive Rate (TPR). While Vazquez et al.’s method shows strong performance in identifying single-compressed videos, its rule-based approach is insufficient for detecting HEVC double-compressed videos. Conversely, the method by He et al. struggles due to the limited number of IP frames in the experimental setup, resulting in high false detection rates for single-compressed videos.

The method in [23] is affected by the presence of CLAHE- and BLUR-processed videos in our dataset, which introduce slight artifacts into single-compressed videos, particularly in P-frames. These artifacts may resemble inconsistencies typically associated with double compression. As a result, relying solely on IP-frames likely led to an increase in false positives.

On the other hand, although the method by [9] takes B-frames into account, it was originally designed for AVC. There are significant differences in encoding strategies between AVC and HEVC. For example, HEVC introduces a merge mode that allows the reuse of motion information, such as motion vectors, reference picture indices, and prediction directions, without explicit signaling. Due to this structure, skip mode behavior, which served as a useful cue for detecting double compression in AVC, becomes less visible in HEVC, thereby reducing the method’s sensitivity to double-compression artifacts in the HEVC setting. To complement our main results and provide additional insight into training stability, we include a histogram of training and validation accuracies across all runs in the Appendix A.

### 5.3. Performance Evaluation for Different Bitrate Combinations

**Figure 4:**
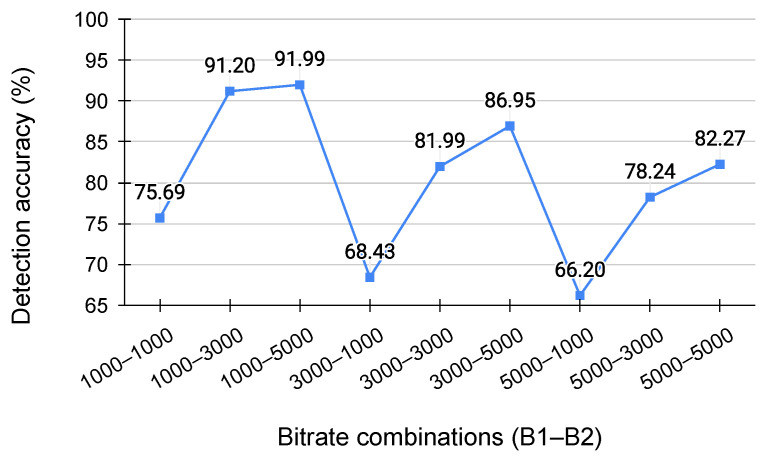
Detection accuracy for each bitrate combination.

To assess the impact of different bitrate combinations, we evaluated the classifier on a test dataset of double-compressed videos encoded with nine distinct bitrate scenarios. Each scenario included 108 single- and double-compressed videos, with the second bitrate consistent across all samples. Figure 4 displays test accuracy for each bitrate scenario, with the X-axis representing bitrate scenarios and the Y-axis showing accuracy.

When the first bitrate (B1) is smaller than the second (B2), the first compression is stronger, leaving residual anomalies in the RI-frames. A larger bitrate difference (B1–B2) improved the accuracy of double-compression detection. On the other hand, when (B1) is larger than (B2), the second compression is stronger, overwriting the anomalies in the RI-frames, and a larger bitrate difference (B1–B2) tended to reduce detection accuracy. When (B1) and (B2) are the same, lower compression strength in (B2) resulted in higher accuracy. These results are generally consistent with the findings discussed in [9], where similar trends were observed in double-compression detection methods.

### 5.4. Performance Evaluation for Different GOP Size Combinations

**Figure 5:**
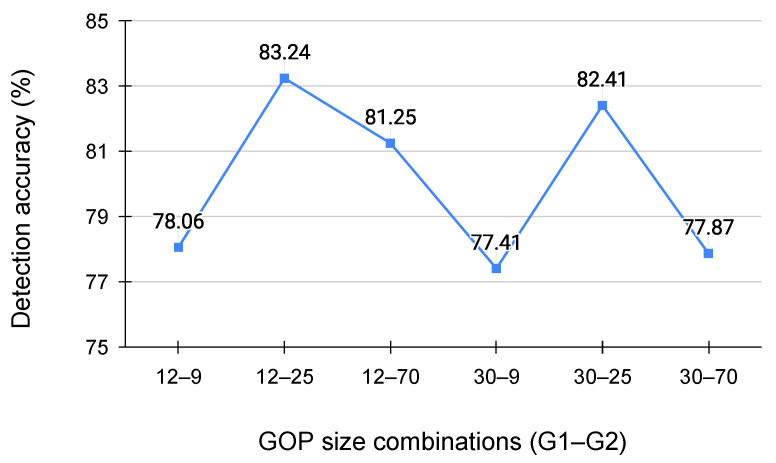
Detection accuracy for each GOP size combination.

We evaluated the classifier’s performance across six different GOP size scenarios to analyze the impact of GOP configuration on double-compression detection. The test dataset consisted of subsets for each GOP size containing 108 single- and double-compressed videos, with a consistent second GOP size. Figure 5 illustrates the results, with the X-axis showing GOP scenarios and the Y-axis indicating test accuracy.

According to the figure, the scenario where the first GOP size (G1) is 12 and the second GOP size (G2) is 25 achieved the highest accuracy. In contrast, the scenario with G1 = 30 and G2 = 9 showed the lowest accuracy. The variation in accuracy is related to the consistent extraction of IP and IB frames, regardless of sub-GOP size.

Table 6 shows the number of IP and IB frames extracted from 500 video frames for each sub-GOP across the six GOP scenarios, along with their sum and standard deviation. In the high-accuracy scenarios of (G1, G2) = (12, 25) and (G1, G2) = (30, 25), the standard deviations of the extracted IP and IB frames are smaller, indicating stable feature vector extraction regardless of sub-GOP size.

### 5.5. Performance Evaluation for Different Amounts of Frames

A test dataset was prepared with double-compressed videos with various amount of frames (500, 400, 300, 200, and 100 frames) to explore how this aspect affects classifier performance. Each subset included 324 single- and double-compressed videos. Table 7 shows that test accuracy goes from an accuracy of 79.72% with 500 frames to a value of 76.56% with 100 frames, suggesting minimal impact of frame size on overall performance. Therefore, while having more frames is ideal, the classifier still maintains adequate performance with fewer frames, making it applicable in scenarios where the number of frames is limited.

## 6. Conclusions

This study proposes a novel classifier designed to detect double compression in HEVC videos by extracting and analyzing encoding information from re-compressed videos that utilize B-frames. By leveraging a reduced set of features and the ability of the Bi-LSTM classifier to capture their temporal evolution, the classifier achieves a significant improvement in detection accuracy, notably outperforming existing models. To our knowledge, this is the first study that specifically addresses the challenge of detecting double compression in HEVC videos containing B-frames, filling a crucial gap in the current literature.

The results demonstrate that spatiotemporal inconsistencies introduced by re-encoding can be effectively captured by encoding-based features and modeled using a hybrid architecture combining convolutional and recurrent layers. However, the method has certain limitations. For instance, while the classifier was evaluated on videos ranging from 100 to 500 frames and showed stable accuracy across this range, it has not been tested on longer sequences exceeding 500 frames, which may appear in real-world content. In addition, all tests were conducted at a fixed resolution, limiting generalizability to videos with varying spatial characteristics. Moreover, this study did not examine other encoding elements, such as in-loop filters like Sample Adaptive Offset and the deblocking filter.

Another limitation is the restriction to closed GOP structures. This design choice was made to simplify the analysis of frame transitions and ensure consistency in feature extraction by preventing frame dependencies across GOP boundaries. However, open GOPs are frequently used in real-world encodings and introduce additional complexity due to cross-GOP prediction. Future work will address this by extending the method to handle open GOPs, further enhancing its practical applicability.

To further enhance robustness under real-world conditions, future work may incorporate additional types of visual distortions beyond blur and lighting variation. Environmental degradations, such as snow and haze, can affect encoding behavior and the visibility of double compression traces. Relevant methods such as DBLRNet [28] (motion blur), DDMSNet [29] (snow), and MB-TaylorFormer V2 [30] (haze) may serve as useful references for building realistic degradation pipelines.

In future research, we aim to extend the classifier’s applicability to more realistic scenarios, specifically to detect double compression in HEVC videos where both the first and second compressions include B-frames. This extension will further enhance forensic applicability by identifying encoding inconsistencies, even in cases where both compressions contain B-frames. Furthermore, we will consider robustness across various video qualities and encoding settings, ensuring adaptability to changes in bitrate and other compression parameters.

## Figures and Tables

**Table 1:** Feature Vector Composition.

Feature Category	Feature Item
Frame Type	I: 100, B: 010, P: 001
CU Size	64 × 64, 32 × 32, 16 × 16, 8 × 8
CU Type	Intra (PI/BI)
	Skip (PS/BS)
	Merge (PM/BM)
	Past MV (Zero) (PVZ/-)
	Past MV (Non Zero) (PVNZ/BP)
	Bi-directional MV: BB
	Future MV: BF
Luminance Directions	0, 1, 9, 10, 11, 25, 26, 27
Chrominance Directions	0, 1, 9, 10, 34, 36

**Table 2:** Ablation study of feature sets. The evaluation metrics used are feature vector dimension (DIM), classification accuracy (ACC, %), and standard deviation (SD).

Feature Vectors	DIM	ACC	SD
Frame Type + CU Type + QP + CU Size + LUMA + CHROMA	80	79.86	1.28
Frame Type + CU Type + CU Size + LUMA + CHROMA	28	80.06	1.16
Frame Type + CU Type + QP + LUMA + CHROMA	76	76.86	1.99
Frame Type + CU Type + QP+ CU Size + CHROMA	72	78.72	1.77
Frame Type + CU Type + QP+ CU Size + LUMA	74	77.58	1.70

**Table 3:** Network layer configuration.

Normalized Feature Vectors: (500 Frames, 28 Dimensions)					
**Layer**	**Input Size**	**Output Size**	**Kernel Size**	**Stride**	**Activation**
Conv1D-1	(500, 28)	(500, 64)	3	1	ReLU
Conv1D-2	(500, 64)	(500, 128)	3	1	ReLU
Bi-LSTM	(500, 128)	(500, 64)	N/A	N/A	None
Linear	(500, 64)	(500, 1)	N/A	N/A	Sigmoid

**Table 4:** Encoding parameters for dataset creation.

Parameters	Single Compression	Double Compression
Encoder	x265	x265
Decoder	HM16.25	HM16.25
Resolution	1280 × 720	1280 × 720
YUV videos (O)	43	43
YUV videos (C)	43	43
YUV videos (B)	43	43
Dataset (O, C, B)	43	43
1st Bitrate	B2{1000, 3000, 5000}	B1{1000, 3000, 5000}
1st GOP	G2{9, 25, 70}	G1{12, 30}
2nd Bitrate	-	B2{1000, 3000, 5000}
2nd GOP	-	G2{9, 25, 70}
Sub-GOP	SG{3, 5, 7}	SG{3, 5, 7}
Videos per Dataset	81 (=3 × 3 × 3 × 3)	486 (=3 × 3 × 2 × 3 × 3 × 3)

**Table 5:** Performance for double compression detection (%). The evaluation metrics used are true positive rate (TPR), true negative rate (TNR), accuracy (ACC), and the standard deviation (SD).

Method	TNR	TPR	ACC	SD
The proposed method	78.07	82.05	80.06	1.16
Vazquez et al. [9]	77.71	45.12	61.42	1.81
He et al. [23]	33.51	82.07	57.79	0.60

**Table 6:** The number of RI-frames and the standard deviation for each GOP size and sub-GOP.

GOP (G1, G2)	Type	Sub-GOP	Num. of IP Frames	Num. of IB Frames
(12, 9)	Data	3	0	28
	Data	5	15	13
	Data	7	0	28
	SUM	–	15	69
	SD	–	8.66	8.66
(12, 25)	Data	3	8	32
	Data	5	8	32
	Data	7	5	35
	SUM	–	21	99
	SD	–	1.73	1.73
(12, 70)	Data	3	22	18
	Data	5	16	24
	Data	7	10	30
	SUM	–	48	72
	SD	–	6.00	6.00
(30, 9)	Data	3	0	11
	Data	5	5	6
	Data	7	0	11
	SUM	–	5	28
	SD	–	2.89	2.89
(30, 25)	Data	3	3	10
	Data	5	0	13
	Data	7	0	13
	SUM	–	3	36
	SD	–	1.73	1.73
(30, 70)	Data	3	7	7
	Data	5	6	8
	Data	7	2	12
	SUM	–	15	27
	SD	–	2.65	2.65

**Table 7:** Performance for double compression detection for each frame size (%).

Frame Size	TNR	TPR	Acc
500	78.12	81.33	79.72
400	76.26	79.82	78.04
300	76.48	79.32	77.90
200	77.81	79.48	78.64
100	72.75	80.37	76.56

**Figure A1:**
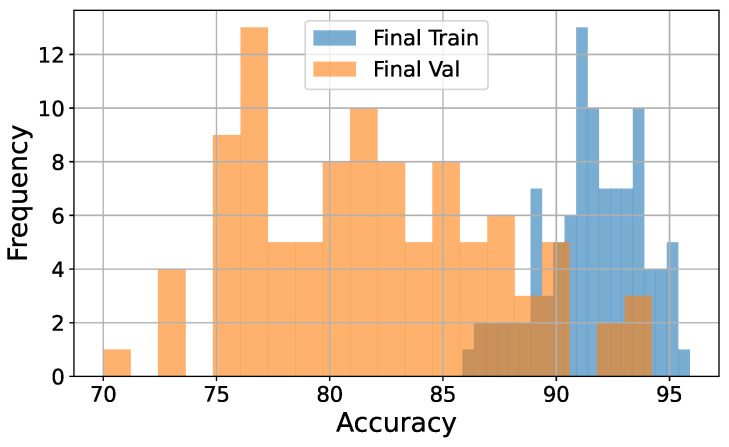
Histogram of final training and validation accuracies collected from 100 folds, obtained by repeating 10-fold cross-validation ten times.

## Data Availability

Data supporting the findings of this study are available on reasonable request.

## Abbreviations

The following abbreviations are used in this manuscript:

Bi-LSTM	A bidirectional Long Short-Term Memory
GOP	Group of Pictures
RI-frames	Relocated I-frames
DCT	Discrete Cosine Transform
QP	Quantization Parameter
CTU	Coding Tree Unit
CU	Coding Unit
PU	Prediction Unit
TU	Transform Unit
TPR	True Positive Rate
TNR	True Negative Rate
ACC	Accuracy
SD	Standard Deviation

## Appendix A

This appendix provides additional analysis on training stability. Specifically, we present a histogram of final training and validation accuracies collected from a total of 100 folds, obtained by repeating 10-fold cross-validation ten times with different random seeds. Each accuracy value corresponds to the final epoch selected by early stopping in each run. Thus, the figure reflects the distribution of fully trained model performance across different random seeds.

The analysis of the distributions reveals the absence of extreme outliers, suggesting that the model consistently converges to similar accuracy levels across different folds and random seeds. This indicates a relative insensitivity to both initialization and fold assignment. Although validation accuracies are, as expected, somewhat lower than those observed on the training set, the two distributions are not markedly different, indicating a limited degree of overfitting and supporting the generalization capability of the model.
